# Longitudinal changes in the inferior cerebellar peduncle and lower limb motor recovery following subcortical infarction

**DOI:** 10.1186/s12883-021-02346-x

**Published:** 2021-08-17

**Authors:** Gang Liu, Yaomin Guo, Chao Dang, Kangqiang Peng, Shuangquan Tan, Chuanmiao Xie, Shihui Xing, Jinsheng Zeng

**Affiliations:** 1 Department of Neurology, The First Affiliated Hospital, Sun Yat–Sen University; Guangdong Provincial Key Laboratory for Diagnosis and Treatment of Major Neurological Diseases, National Key Clinical Department and Key Discipline of Neurology, No. 58, Zhongshan Road 2, Guangzhou, Guangdong China; 2 Guangdong-Hong Kong-Macao Greater Bay Area Center for Brain Science and Brain-Inspired Intelligence, Guangzhou, Guangdong China; 3 Department of Medical Imaging, Sun Yat–Sen University Cancer Center, State Key Laboratory of Oncology in Southern China, Collaborative Innovation Center for Cancer Medicine, Guangzhou, Guangdong China

**Keywords:** Diffusion tensor imaging, Fugl-Meyer, Inferior cerebellar peduncle, Lower limb, Subcortical infarction

## Abstract

**Background:**

The cerebellum receives afferent signals from spinocerebellar pathways regulating lower limb movements. However, the longitudinal changes in the spinocerebellar pathway in the early stage of unilateral supratentorial stroke and their potential clinical significance have received little attention.

**Methods:**

Diffusion tensor imaging and Fugl-Meyer assessment of lower limb were performed 1, 4, and 12 weeks after onset in 33 patients with acute subcortical infarction involving the supratentorial areas, and in 33 healthy subjects. We evaluated group differences in diffusion metrics in the bilateral inferior cerebellar peduncle (ICP) and analyzed the correlation between ICP diffusion metrics and changes to the Fugl-Meyer scores of the affected lower limb within 12 weeks after stroke.

**Results:**

Significantly decreased fractional anisotropy and increased mean diffusivity were found in the contralesional ICP at week 12 after stroke compared to controls (all *P* < 0.01) and those at week 1 (all *P* < 0.05). There were significant fractional anisotropy decreases in the ipsilesional ICP at week 4 (*P* = 0.008) and week 12 (*P* = 0.004) compared to controls. Both fractional anisotropy (r_s_ = 0.416, *P* = 0.025) and mean diffusivity (r_s_ = -0.507, *P* = 0.005) changes in the contralesional ICP correlated with changes in Fugl-Meyer scores of the affected lower limb in all patients.

**Conclusions:**

Bilateral ICP degeneration occurs in the early phase of supratentorial stroke, and diffusion metric values of the contralesional ICP are useful indicators of affected lower limb function after supratentorial stroke.

**Supplementary Information:**

The online version contains supplementary material available at 10.1186/s12883-021-02346-x.

## Background

The cerebellum receives afferent signals from both the sensorimotor cortex and the muscles regulating limb movements via the cortico-ponto-cerebellar and spinocerebellar pathways, respectively [1, 2]. The cortico-ponto-cerebellar tract transfers efferent motor signals from the sensorimotor cortex to the contralateral cerebellum via the middle cerebellar peduncle [1]. The spinocerebellar tract, especially the dorsal spinocerebellar tract (DSCT), mainly conveys somatosensory information from the spindles and Golgi tendon organs of the ipsilateral trunk and limb muscles and enters the cerebellum via the inferior cerebellar peduncle (ICP) [3]. Secondary degeneration in the contralesional middle cerebellar peduncle after supratentorial stroke has been widely reported in diffusion tensor imaging (DTI) studies and is associated with the severity of hemiplegic limb function after stroke and proposed as a crucial mechanism of crossed cerebellar diaschisis [4, 5]. However, the pattern of ICP changes after supratentorial stroke and their relationship with post-stroke changes of motor function have been rarely reported.

Recently, Kim et al. [6] found a secondary degeneration characterized by reduced fractional anisotropy (FA) and elevated mean diffusivity (MD) in the contralesional ICP in 23 patients with subacute middle cerebral artery (MCA) stroke, a change associated with patients’ ambulatory and lower limb (LL) function. They believed that insufficient peripheral proprioceptive stimulation to the cerebellum due to LL weakness might result in secondary changes in the spinocerebellar pathway, but they could not fully determine whether FA reduction in the contralesional ICP was affected by hemiplegic muscles, a hypofunctional cerebellum, or both, due to the retrospective and cross-sectional design of their study. Therefore, longitudinal studies are necessary to clarify the possible mechanism of contralesional ICP degeneration after supratentorial ischemic stroke. In addition, Li et al. [7] reported that structural reorganization of the ipsilesional ICP could be induced via repetitive transcranial magnetic stimulation of the ipsilesional primary motor cortex in patients with acute subcortical infarction involving the MCA territory, suggesting a possible direct connection between the ICP and the ipsilateral primary motor cortex. Studies with probabilistic fiber tracking further confirmed that, in addition to a 100 % connectivity between the ICP and the anterior lobe of the cerebellum, especially lobules IV–V, the ICP also had high connectivity with ipsilateral sensory-motor related cerebral cortices [8]. Nevertheless, whether supratentorial stroke lesions can also cause secondary damage in the ipsilesional ICP due to a connectivity disruption between the ICP and sensory-motor cortices remains unclear.

Currently, the amplitude of low-frequency fluctuation (ALFF), a method to measure the signal intensity in low-frequency (e.g., 0.01–0.08 Hz) oscillations of spontaneous neural activity during resting state [9], is considered to correlate with field potential activity in regional brain areas [10]. Furthermore, ALFF can be used as an index to examine changes in neural function [11]. This approach has been widely applied for evaluating local brain function in healthy participants [12] and patients with neurological diseases [13, 14]. Moreover, it has been reported that ALFF was altered under resting state in stroke patients and that the ALFF values in sensorimotor cortices were related to the severity of motor deficits [15, 16]. In this study, we hypothesized that secondary degeneration might occur in the bilateral ICP in the early phase of supratentorial ischemic stroke, but the mechanisms underlying bilateral ICP degeneration may differ. Insufficient peripheral proprioceptive stimulation due to LL weakness may lead to the degeneration of the contralesional ICP after supratentorial stroke, but secondary damage in the ipsilesional ICP may be attributed to Wallerian degeneration, a well-known phenomenon defined as anterograde degeneration of a nerve tract distal to an ischemic injury [17–19]. Furthermore, diffusion parameter values in the contralesional ICP degeneration may be indicative of poorer LL function. In order to test our hypothesis, DTI combined with ALFF was used to assess the microstructural integrity of the ICP and functional changes of the anterior lobe of the cerebellum (lobules IV–VI), respectively, during a 12-week follow-up after acute subcortical infarction involving the supratentorial regions. We also analyzed the correlation between changes to these indices and improvements in the motor function of the affected LL.

## Methods

### Participants

**Fig. 1:**
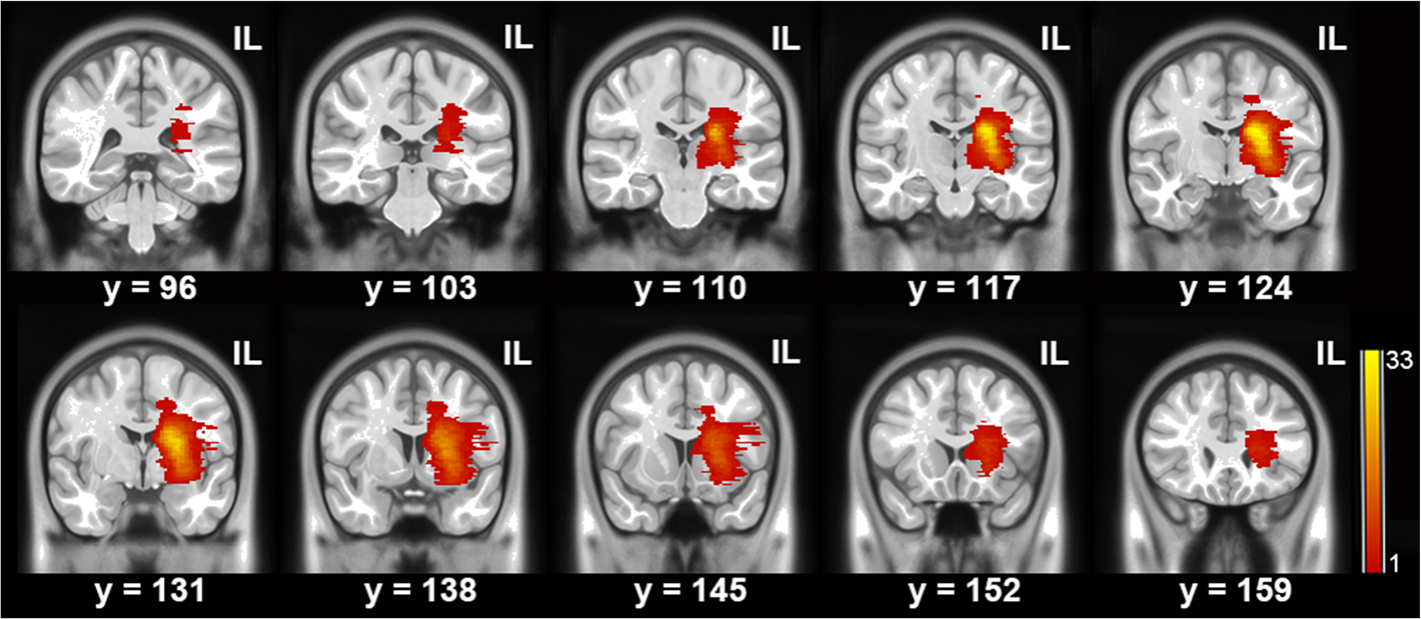
Lesion maps of the patients. All lesions are aligned to the same hemisphere for visualization. Colors represent the number of patients with a lesion at the given location. IL = ipsilesional side

**Fig. 2:**
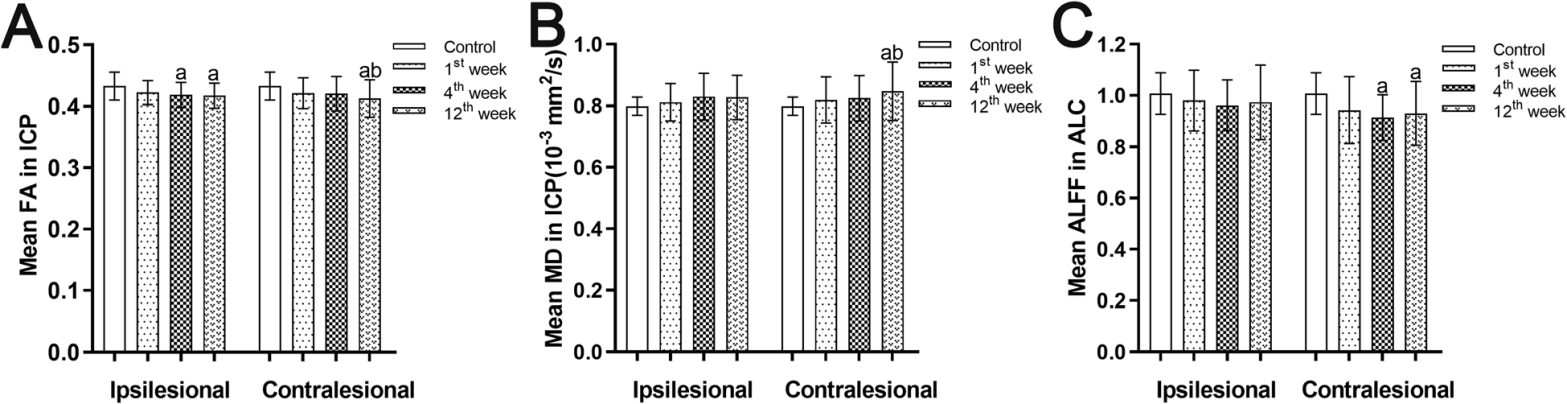
The diffusion parameters and ALFF values differences between groups. Bar charts represent the mean FA (**A**) values and MD (**B**) values of the ICP and the mean ALFF (**C**) values of the ALC in patients at different time points after stroke and in controls. The error bars represent the standard deviation of the mean. ALC, anterior lobe of cerebellum; ALFF, amplitude of low-frequency fluctuation; FA, fractional anisotropy; ICP, inferior cerebellar peduncle; MD, mean diffusivity. ^a^*P* < 0.05, compared with the controls, ^b^*P* < 0.05, compared with the first week

This research was approved by the First Affiliated Hospital of Sun Yat-Sen University ethics committee. Written informed consent was obtained from each participant. We applied the following inclusion criteria: (1) a first unilateral supratentorial subcortical infarct (< 7 days) (Fig. 1); (2) no intra-cranial or extra-cranial artery occlusion on magnetic resonance angiography confirmed by either ultrasound or magnetic resonance angiography; (3) baseline scores of < 34 on the LL component of the Fugl–Meyer (FM-LL); (4) aged 18–75 years; and (5) patients receiving routine rehabilitation therapies, as reported earlier [16, 20–22]. The exclusion criteria were as follows: (1) medical implants contraindicated for cerebral magnetic resonance imaging (MRI); (2) additional neurological diseases other than stroke; (3) traumatic brain injury or psychiatric disease or alcohol abuse; (4) any revascularization therapy; or (5) presence of aphasia or apraxia, or use of any medications likely to affect motor examinations during the follow-up period. We also recruited age- and gender-matched healthy controls.

### Experimental design

Each patient underwent three MRI acquisitions: within the first week (week 1; < 7 days), the fourth week (week 4; 28 ± 4 days), and the 12th week (week 12; 84 ± 4 days) [16, 20–22]. Neurological and FM-LL assessments were performed before the MRI examination at each time point. Controls, however, were examined only once.

### Behavioral assessments

The National Institutes of Health Stroke Scale was used to assess initial neurological deficits. FM-LL scale was administered to evaluate LL motor deficits at 1 week, 4 weeks, and 12 weeks after stroke.

### Image acquisition

MRI was performed using a 3 T MRI system (Tim Trio; Siemens, Erlangen, Germany). High resolution (1 × 1 × 1 mm^3^) 3D T1-weighted images were acquired using MPRAGE, with repetition time (TR) = 2,530 ms, echo time (TE) = 3.45 ms, inversion time = 1,100 ms, flip angle = 7°, field of view = 256 × 256 mm^2^, and 192 slices. DTI data were gained using a single-shot echo-planar imaging sequence with TR = 7,000 ms, TE = 91 ms, flip angle = 90°, matrix = 128 × 128, voxel size = 2 × 2 × 3 mm^3^, axial slices = 50, field of view = 256 × 256 mm^2^, non-collinear directions = 64, and b = 1,000 s/mm^2^. Resting-state functional images were collected using an echo-planar imaging sequence with TR = 2,000 ms, TE = 30 ms, flip angle = 90°, field of view = 220 × 220 mm^2^, voxel size = 3.44 × 3.44 × 3 mm^3^, averages = 1, and axial slices = 33.

### DTI and resting-state functional data preprocessing

DTI data preprocessing was performed using the Pipeline for Analyzing Brain Diffusion Images toolkit (PANDA, http://www.nitrc.org/projects/panda/) [23] and FSL (http://fsl.fmrib.ox.ac.uk/fsl) [24]. The detailed preprocessing procedures can be obtained from our previous reports [16, 20–22]. The functional MRI images were preprocessed using Data Processing & Analysis for Brain Imaging (DPABI; http://rfmri.org/DPABI), which is based on Resting-state Data Analysis Toolkit (REST; http://www.restfmri.net) and Statistical Parametric Mapping (SPM12; http://www.fil.ion.ucl.ac.uk/spm). Briefly, preprocessing procedures included the first 10 volumes’ removal, slice timing and motion correction, the transformation from individual space to Montreal Neurological Institute space, spatial smoothing with a Gaussian kernel of 4 × 4 × 4 mm^3^, and band-pass filtering (0.01–0.08 Hz). Participant data were excluded if they met the head motion criteria, which included head motion > 2 mm translation or a 2° rotation in any direction. For the ALFF calculation, the time courses were first converted to the frequency domain using a fast Fourier transform. The square root of the power spectrum was then computed and averaged across 0.01–0.08 Hz at each voxel. This averaged square root was considered as the ALFF [12–14]. To reduce the global effects of variability across participants, the ALFF of each voxel was divided by the global mean ALFF value obtained for each participant. Lesion volume was calculated by manually drawing stroke lesion on the T1-weighted images in individual space [25], and the corticospinal tract (CST) volume was measured as previously described [26, 27] at week 1 after stroke. The details on CST volume evaluation are provided in the Supplemental Methods.

### Regions of interest (ROIs) analysis

For ROIs analysis, we selected the bilateral ICP and the anterior lobe of the cerebellum (lobules IV–VI). The bilateral ICP was extracted from the Johns Hopkins University white matter tractography atlas (JHU-ICBM-DTI-81-WMPM-90p) [28] and the bilateral anterior lobe of the cerebellum from the Automated Anatomical Labeling atlas. Averaged FA and MD values for each ICP were obtained for each participant using the corresponding normalized diffusion metric maps. The mean ALFF values of each anterior lobe of the cerebellum were acquired for each participant using the individual ALFF maps.

### Statistical analysis

Categorical variables were compared using the Pearson χ2 or Fisher exact tests (when the expected number was ≤ 5). Age was compared between the patient group and the control group using the independent *t*-test after normality testing by the Shapiro-Wilk test. In the patient group, FM-LL scores obtained at multiple time points were compared using the paired-samples Wilcoxon signed-rank test. Analyses of variance with repeated measures were employed to compare absolute values within ROIs between time points, and a *post hoc* analysis was conducted based on the Bonferroni correction for multiple testing. A Spearman’s partial correlation analysis was used to determine correlations between changes in FM-LL scores and changes in ICP diffusion parameter values when adjusting for age, gender, lesion volume, and CST volume_ipsilesional_/CST volume_contralesional_ ratio as covariates, which was defined as the difference between FM-LL scores or diffusion parameter values at baseline and 12 weeks. All tests were two-sided, and *P* < 0.05 was considered significant. The FA and MD values of the ICP and the ALFF values of the anterior lobe of the cerebellum at each time point were compared with those of controls using an independent *t*-test with a corrected *P* value threshold (corrected *P*_threshold_ = 0.05/3). All analyses were conducted using SPSS 16.0 for Windows (SPSS, Chicago, IL, USA).

## Results

### Participant characteristics and behavioral assessment

In total, 33 patients (14 women and 19 men, mean age: 53.4 years) and 33 healthy participants (controls) (14 women and 19 men, mean age: 53.7 years) were included in the study. The demographic and clinical characteristics of the groups are detailed in Table 1 and Supplemental Table I. No significant differences in age, sex, and vascular risk factors except for hypertension were found between groups. In the patient group, FM-LL scores at week 4 and week 12 were significantly higher than those at week 1 (all *P* < 0.001, Cohen’s *d* = 3.859), while the FM-LL scores at week 12 were significantly higher than those at week 4 (*P* < 0.001, *d* = 3.441).

**Table 1:** Demographic and clinical characteristics of the groups

	Patient group (*n* = 33)	Control group (*n* = 33)
Age, years (Mean ± SD)	53.4 ± 1.81	53.7 ± 2.32
Women, n (%)	14 (42.4)	14 (42.4)
Hypertension, n (%)	19 (57.6)	11 (33.3)^*^
Diabetes mellitus, n (%)	11 (33.3)	7 (21.2)
Hypercholesterolemia, n (%)	7 (21.2)	5 (15.2)
Tobacco users, n (%)	4 (12.1)	4 (12.1)
Lesion in left hemisphere, n (%)	17 (51.5)	
Median lesion volume (range; mL)	5.9 (0.9–28.5)	
Median NIHSS at week 1 (range)	9 (2–18)	
Median FM-LL at week 1 (range)	9 (00–33)	
Median FM-LL at week 4 (range)	27 (00–34)^†^	
Median FM-LL at week 12 (range)	32 (11–34)^†#^	

Empty cells indicate no assessment. FM-LL indicates Fugl-Meyer score of lower limb; NIHSS, National Institutes of Health Stroke Scale. *P < 0.05, compared with the patient group. ^†^*P* < 0.001, compared with week 1; ^#^*P* < 0.001, compared with week 4

### Dynamic changes in DTI-derived metrics and ALFF after stroke

The results of the comparison of the DTI-derived metrics and ALFF are shown in Fig. 2. Significantly decreased FA and increased MD were found in the contralesional ICP at week 12 after stroke compared with controls (FA, *P* = 0.003, *d* = 0.753; MD, *P* = 0.008, *d* = 0.692) and with values recorded at week 1 (FA, *P* = 0.035, *d* = 0.543; MD, *P* = 0.025, *d* = 0.584). There were significant FA decreases but not MD increases in the ipsilesional ICP at week 4 (*P* = 0.008, *d* = 0.674) and week 12 (*P* = 0.004, *d* = 0.729) compared to controls, but no significant differences in the FA and MD of the ipsilesional ICP between time points. No significant correlations between lesion volumes, CST volume_ipsilesional_/CST volume_contralesional_ ratios, and FA and MD changes of the contralesional ICP during a 12-week follow-up were observed respectively.

The results of ALFF analysis revealed that compared with the control group, the patient group exhibited significant decreases in the mean ALFF values of the contralesional anterior lobe of the cerebellum from week 4 (*P* < 0.0001, *d* = 1.108) to week 12 (*P* = 0.004, *d* = 0.74) after stroke, but such changes were not found in the ipsilesional anterior lobe of the cerebellum. The mean ALFF values of the bilateral anterior lobe of the cerebellum did not significantly differ between time points.

### Correlational analyses between motor assessments and DTI-derived metrics

**Fig. 3:**
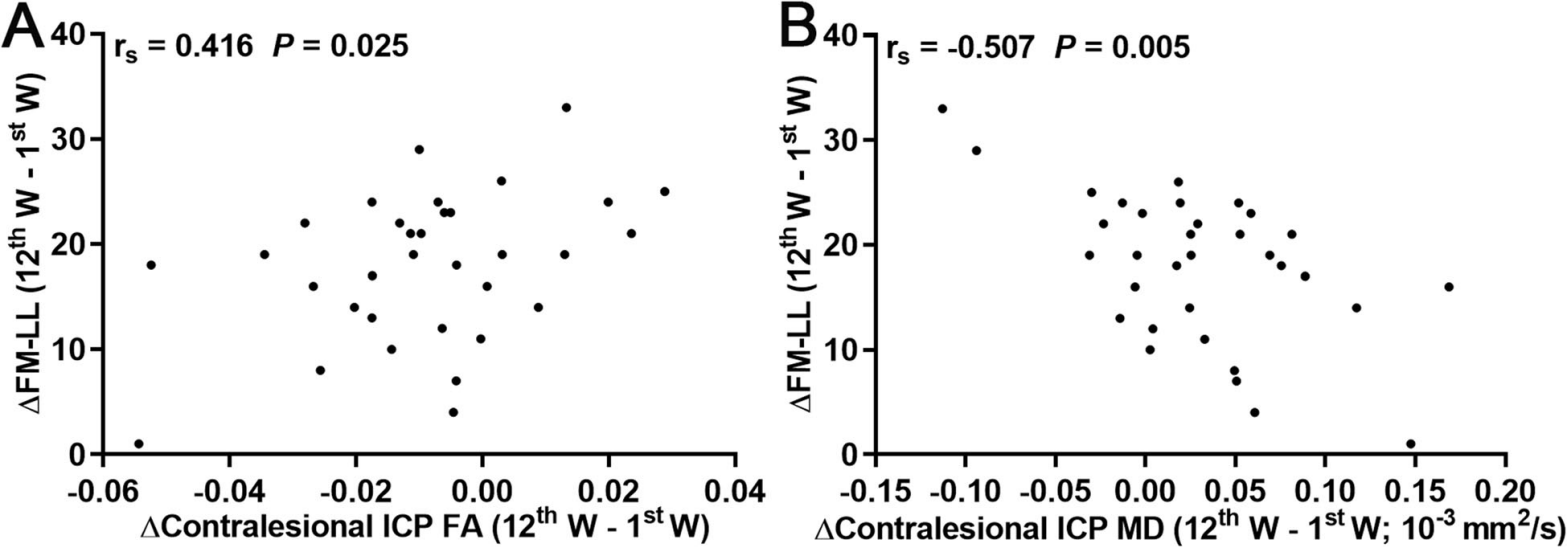
Spearman correlation maps. Spearman correlation plots for change in FM-LL scores (y-axis), as well as the change in FA (**A**) and MD (**B**) values of ICP (x-axis). ΔFM-LL scores or FA and MD of the contralesional ICP are values measured at week 12 after deducting values measured at week 1. FA, fractional anisotropy; FM-LL, Fugl-Meyer score of lower limb; ICP, inferior cerebellar peduncle; MD, mean diffusivity; W, week. r_s_ = Spearman correlation coefficient

As shown in Fig. 3, Spearman’s partial correlation analysis revealed that both FA (*r*_*s*_ = 0.416, *P* = 0.025) and MD (*r*_*s*_ = -0.507, *P* = 0.005) changes in the contralesional ICP correlated with changes in FM-LL scores across patients when adjusting for age, gender, lesion volume, and CST volume_ipsilesional_/CST volume_contralesional_ ratio as covariates.

## Discussion

In this study, we found that patients with acute subcortical infarction involving the supratentorial areas exhibited significantly decreased FA in the bilateral ICP and reduced ALFF in the contralesional anterior lobe of the cerebellum post-stroke. The FA and MD changes in the contralesional ICP found within a 12-week follow-up period correlated with FM-LL score changes of the affected limb across patients. These findings supported our hypothesis that (1) secondary degeneration occurred in the bilateral ICP in the early phase of supratentorial ischemic stroke, and (2) diffusion parameter values in the contralesional ICP were useful indicators of affected LL function.

The cerebellum receives afferent signals from both the sensorimotor cortex and the muscles regulating limb movements via the cortico-ponto-cerebellar and spinocerebellar pathways, respectively [1, 2]. There are two kinds of spinocerebellar tracts from the LL: ventral and dorsal spinocerebellar tracts. The DSCT transmits proprioceptive information of the LL muscles to the ipsilateral cerebellum via the ICP [3]. Therefore, the DSCT is of particular importance in the control of posture and locomotion [29, 30]. It is challenging to evaluate the DSCT integrity in a living human brain since it is very small and indistinguishable from adjacent structures using conventional neuroimaging [6]. Nevertheless, DTI enables the evaluation of the microstructural integrity of the ICP [31]. Therefore, we focused on LL function based on previous investigations that reported the ICP as a key structure involved in the control of body posture and ambulation [32–34]. Significant FA decreases and MD increases at 12 weeks after stroke were detected in the contralesional ICP, indicating a secondary degeneration of the contralesional ICP far from the stroke lesion, progressively deteriorating with time, which can be clearly and quantitatively detected by DTI. Kim et al. [6] found a marked FA reduction and MD increase in the contralesional ICP at an earlier stage (median: 27 days) after stroke in the patient group compared with the control group. The discrepancy in the time of diffusion parameter changes between this study and the study conducted by Kim et al. may be due to experimental design differences. In their study, only patients with modified Rankin Scale score ≥ 3 were recruited, while in the present study, patients with less severe deficits in LL function were also included. Therefore, our results can be readily generalized to a wider patient population with supratentorial stroke. We found that changes in both FA and MD in the contralesional ICP significantly correlated with changes in FM-LL scores during a 12-week follow-up period, suggesting that the greater extent of disruption in the microstructural integrity of the contralesional ICP was indicative of poorer LL function after stroke. The integrity of the CST has been correlated with LL function using the FM assessment of the lower extremities [35]. However, recent studies have indicated that CST integrity, as evaluated by the CST lesion load, was only weakly associated with LL function [36, 37] and did not accurately predict a proportional recovery of the lower extremities after stroke [38]. Therefore, the LL motor recovery following stroke is insufficiently explained by the integrity of the CST alone. In addition, we failed to find any correlations between lesion volumes, CST volume_ipsilesional_/CST volume_contralesional_ ratios, and changes of FA and MD of the contralesional ICP during the follow-up period. Therefore, our result that LL motor function was also associated with ICP integrity is noteworthy.

Kim et al. [6] considered that insufficient peripheral proprioceptive stimulation to the cerebellum from a weak LL might result in secondary degeneration in the contralesional spinocerebellar pathway, but they could not fully determine whether FA reduction in the contralesional ICP was affected by hemiplegic muscles, a hypofunctional cerebellum, or both, due to the retrospective and cross-sectional design of their study. We found that ALFF in the contralesional anterior lobe of the cerebellum reduced sharply at week 4 but increased somewhat at week 12 after onset, which is consistent with the findings reported by Kim et al. [6]. They found that a high percentage of the patients with a lower FA of the contralesional ICP exhibited contralesionally reduced cerebellar function in the subacute phase of supratentorial stroke using ^99m^Tc-hexamethylpropyleneamineoxime SPECT technique. They assumed that ICP degeneration might be responsible for the decreased cerebellar function. However, in this study, we believed that it is impossible for ICP degeneration to contribute to reduced cerebellar function because ICP degeneration occurred after cerebellar function declined. Insufficient peripheral proprioceptive stimulation due to LL weakness may lead to a hypofunctional cerebellum and the degeneration of the contralesional ICP after supratentorial stroke, but whether a hypofunctional cerebellum also contributes to the degeneration in the contralesional ICP after a supratentorial stroke needs to be verified by future studies.

In the present study, patients also exhibited a degeneration characterized by reduced FA in the ipsilesional ICP in the early phase of supratentorial ischemic stroke. The mechanisms of secondary degeneration in the ipsilesional ICP should be different from those in the contralesional ICP because the motor function of the ipsilesional LL is not affected. Studies with probabilistic fiber tracking confirmed that the ICP was not only connected to the anterior lobe of the cerebellum but also with ipsilateral sensory-motor related cerebral cortices. In the present study, we also found that the patients exhibited a disruption of the ipsilesional connectivity between the ICP and cerebral cortices due to the ischemic lesion in the acute stage of stroke using a DTI-based deterministic fiber tracking algorithm (see Supplemental Fig. I). Therefore, we believe that the disruption of structural integrity in the ipsilesional ICP may be attributed to Wallerian degeneration, a well-known phenomenon defined as anterograde degeneration of a nerve tract distal to an ischemic injury [17–19].

A major limitation of this study is that the ICP selected in our study contains multiple fiber tracts connected to the cerebellum. Therefore, FA and MD of the ICP were not specific to the DSCT, mainly because of its small size and the difficulty to differentiate it from adjacent tracts due to the limited resolution of the DTI sequence. Future studies should be conducted focusing on the pattern of changes of individual tracts such as DSCT following supratentorial stroke, which may refine our findings. In addition, although the ICP also includes the cuneocerebellar tract, which conveys somatosensory signals from the ipsilateral upper limb muscle to the cerebellum, the role of ICP degeneration in the functional upper limb affectation after supratentorial stroke remains unknown because of a lack of neuroimaging basis for the clinic-anatomic association between upper limb function and ICP integrity. Finally, the relatively small sample size of our study is another limitation.

## Conclusions

Our results provide evidence that secondary degeneration occurs in the bilateral ICP following an acute subcortical infarct involving supratentorial areas and that a greater extent of microstructural disruption in the contralesional ICP is indicative of a poorer LL function, but the mechanisms of bilateral ICP degeneration may be different. The findings of this study will improve our understanding of the mechanisms underlying the action of neuromodulation techniques, including peripheral nerve and cerebellar stimulation. In addition, we expect the ICP to be a potential treatment target of neuromodulation strategies for improving affected LL function after stroke.

## Supplementary Information

**Additional file 1.**

## Data Availability

The datasets in the current study are available from the corresponding author on reasonable request.

## Abbreviations

ALFF: Amplitude of low-frequency fluctuation
ALC: Anterior lobe of cerebellum
CST: Corticospinal tract
DSCT: Dorsal spinocerebellar tract
DTI: Diffusion tensor imaging
FA: Fractional anisotropy
FM-LL: Lower limb component of the Fugl–Meyer
ICP: Inferior cerebellar peduncle
LL: Lower limb
MCA: Middle cerebral artery
MD: Mean diffusivity
MRI: Magnetic resonance imaging
ROIs: Regions of interest
SPECT: Single-photon emission computed tomography
TE: Echo time
TR: Repetition time
