# Supplementary material for: Longitudinal changes in the inferior cerebellar peduncle and lower limb motor recovery following subcortical infarction
Source: BMC Neurol. 2021 Aug 17;21:320. doi: 10.1186/s12883-021-02346-x (PMC8369783; doi:10.1186/s12883-021-02346-x)
Supplement: Supplementary file 1 — Additional file 1. [file 12883_2021_2346_MOESM1_ESM.docx]

**SUPPLEMENTAL MATERIAL**

**Longitudinal changes in the inferior cerebellar peduncle and lower limb motor recovery following subcortical infarction**

Gang Liu^1^***,** Yaomin Guo^1^*, Chao Dang^1^*, Kangqiang Peng^2^, Shuangquan Tan^1^, Chuanmiao Xie^2^, Shihui Xing^1^, Jinsheng Zeng^1^

^1^ Department of Neurology, The First Affiliated Hospital, Sun Yat–Sen University; Guangdong Provincial Key Laboratory for Diagnosis and Treatment of Major Neurological Diseases, National Key Clinical Department and Key Discipline of Neurology, Guangzhou, Guangdong, China

^2^ Department of Medical Imaging, Sun Yat–Sen University Cancer Center, State Key Laboratory of Oncology in Southern China, Collaborative Innovation Center for Cancer Medicine, Guangzhou, Guangdong, China

**Supplemental Methods**

**Corticospinal tract reconstruction**

Diffusion Toolkit (<http://www.trackvis.org>) and Trackvis (<http://www.trackvis.org>) were used for fiber tractography and reconstruction of corticospinal tract (CST) [1]. Tracking was performed from all voxels inside the brain using the deterministic tract reconstruction with a Fiber Association by Continuous Tracking algorithm (FACT; 35° angular threshold). Then, the regions of interests (ROIs) symmetrically in the left and right sides along the CST pathway at two levels: the pontomedullary junction and the posterior limb of internal capsule were used to reconstruct the CST, which exploits existing anatomical knowledge of tract trajectories [2] and previously described methods [3-5]. According to the size of the brain structure, the 30 mm^2^ and 35 mm^2^ ROIs were manually placed on the individual diffusion weighted imaging cross-referenced with the color FA maps in the pontomedullary junction ventrally and middle of the posterior limb of internal capsule, respectively [5]. The tracts resulting were visually assessed to ensure anatomic accuracy. Finally, values of the CST volume were calculated by multiplying the number of traced voxels by voxel volume.

**Supplemental References**

1. Paldino MJ, Hedges K, Zhang W. Independent contribution of individual white matter pathways to language function in pediatric epilepsy patients. Neuroimage Clin. 2014;6:327-32.

2. Welniarz Q, Dusart I, Roze E. The corticospinal tract: evolution, development, and human disorders. Dev Neurobiol. 2017;77:810-29.

3. Volbers B, Mennecke A, Kästle N, Huttner HB, Schwab S, Schmidt MA, et al. Quantitative corticospinal tract assessment in acute intracerebral hemorrhage. Transl Stroke Res. 2020;10.1007/s12975-020-00850-9.

4. Kim JS, Kim SH, Lim SH, Im S, Hong BY, Oh J, et al. Degeneration of the inferior cerebellar peduncle after middle cerebral artery stroke: another perspective on crossed cerebellar diaschisis. Stroke. 2019;50:2700-7.

5. Zhang M, Lin Q, Lu J, Rong D, Zhao Z, Ma Q, et al. Pontine infarction: diffusion-tensor imaging of motor pathways-a longitudinal study. Radiology. 2015;274:841-50.

**Supplemental Table**

**Table I** Demographic Features and Clinical Characteristics of Patients

| No | Side of Lesion | Volume of Lesion, mL | Stroke Lesion | Risk Factors | FM-LL | | |  | NIHSS |
| --- | --- | --- | --- | --- | --- | --- | --- | --- | --- |
|  |  |  |  |  | Week 1 | Week 4 | Week 12 |  | Week 1 |
| 1 | L | 5.876 | BG, CR | HT | 9 | 26 | 27 |  | 12 |
| 2 | R | 8.928 | BG | HT | 21 | 33 | 33 |  | 6 |
| 3 | L | 17.787 | BG, CR | HT, HC | 11 | 24 | 32 |  | 12 |
| 4 | L | 3.789 | CR | DM | 33 | 34 | 34 |  | 2 |
| 5 | L | 3.757 | CR, IC | HT, DM | 21 | 25 | 28 |  | 7 |
| 6 | R | 6.485 | CR | HT | 16 | 24 | 29 |  | 9 |
| 7 | R | 6.095 | CR, BG | DM | 9 | 27 | 33 |  | 12 |
| 8 | L | 7.653 | CR | HT, DM | 7 | 25 | 31 |  | 11 |
| 9 | R | 27.389 | BG, CR, CS | AF | 7 | 23 | 28 |  | 13 |
| 10 | R | 1.703 | BG, CR | HT, DM, HC | 9 | 23 | 34 |  | 8 |
| 11 | L | 2.302 | BG, CR | HT, DM, HC | 10 | 33 | 33 |  | 9 |
| 12 | L | 3.613 | IC | HT, DM, HC | 24 | 34 | 34 |  | 2 |
| 13 | L | 3.178 | BG, CR | HS | 14 | 33 | 33 |  | 6 |
| 14 | L | 25.798 | BG, CR, CS | None | 0 | 0 | 11 |  | 18 |
| 15 | R | 6.458 | BG, CR | HT | 8 | 10 | 27 |  | 11 |
| 16 | L | 5.052 | BG, CR | HT | 6 | 27 | 30 |  | 10 |
| 17 | R | 28.540 | BG, CR | HT | 3 | 27 | 29 |  | 12 |
| 18 | L | 22.727 | BG, CR, CS | DM | 2 | 18 | 20 |  | 12 |
| 19 | R | 4.587 | IC | None | 16 | 28 | 32 |  | 4 |
| 20 | R | 11.793 | BG | None | 12 | 34 | 34 |  | 4 |
| 21 | R | 6.827 | BG, CR | DM | 0 | 12 | 29 |  | 16 |
| 22 | L | 1.928 | IC | Vasculitis | 0 | 26 | 33 |  | 11 |
| 23 | R | 11.689 | CR | DM, HC | 2 | 16 | 21 |  | 11 |
| 24 | L | 2.312 | BG, CR | HT, HC | 9 | 26 | 25 |  | 7 |
| 25 | L | 5.918 | BG, CR | Vasculitis | 9 | 30 | 32 |  | 7 |
| 26 | R | 3.564 | BG, CR, CS | HS | 13 | 30 | 34 |  | 9 |
| 27 | R | 3.298 | IC | HT | 30 | 34 | 34 |  | 2 |
| 28 | L | 2.494 | THA | HT | 18 | 31 | 32 |  | 8 |
| 29 | R | 3.236 | CR | HT, DM, HS | 6 | 22 | 23 |  | 10 |
| 30 | L | 6.914 | BG, CR | HT | 25 | 33 | 33 |  | 6 |
| 31 | R | 22.939 | BG, CR | HT, HS | 2 | 19 | 24 |  | 10 |
| 32 | L | 6.940 | BG, CR | HT | 11 | 28 | 30 |  | 10 |
| 33 | R | 0.938 | BG, CR | HC | 20 | 32 | 34 |  | 3 |

AF indicates atrial fibrillation; BG, basal ganglia; CR, corona radiata; CS, centrum semiovale; DM, diabetes mellitus; FM-LL, Fugl-Meyer score of lower limb; HC, hypercholesterolemia; HT, hypertension; HS, habitual smoking; IC, internal capsule; L, left; NIHSS, National Institutes of Health Stroke Scale; R, right; THA, thalamus.

**Supplemental Figure**


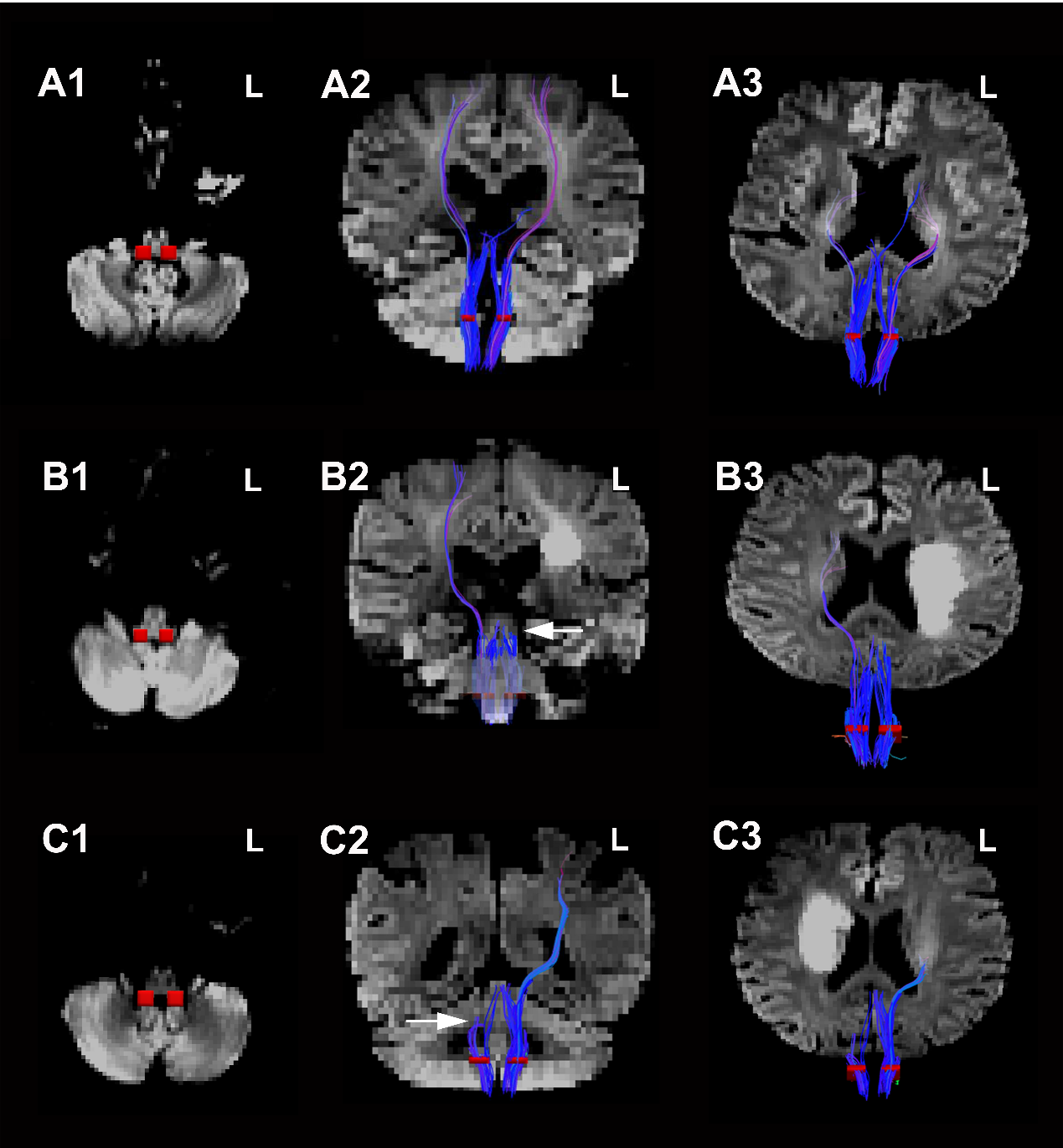


**Fig. I** Examples for deterministic tracking images obtained from a control (A1-A3) and a patient with a left (B1-B3) and a patient with a right (C1-C3) subcortical infarct at the first week after the onset of stroke. (A1-C1) The axial view of the regions of interest representing the bilateral ICP at pontomedullary junction level on the individual diffusion weighted imaging. (A2-C2) The coronal view of left and right fiber tracts connecting the ICP and ipsilateral cerebral cortex overlaid on the individual diffusion weighted imaging. (A3-C3) The axial view of left and right fiber tracts connecting the ICP and ipsilateral cerebral cortex overlaid on the individual diffusion weighted imaging. The white arrows show the disruption of the connectivity between the ICP and ipsilateral cerebral cortex due to the ischemic lesion. ICP, inferior cerebellar peduncle; L, left.
